# Visualizing Commognitive Responsibility Shift in Collaborative Problem-Solving During Computer-Supported One-to-One Math Tutoring

**DOI:** 10.3389/fpsyg.2022.815625

**Published:** 2022-04-07

**Authors:** Jijian Lu, Pan Tuo, Ruisi Feng, Max Stephens, Mohan Zhang, Zhonghua Shen

**Affiliations:** ^1^Jinghengyi School of Education, Hangzhou Normal University, Hangzhou, China; ^2^School of Education, Shaanxi Normal University, Xi’an, China; ^3^Melbourne Graduate School of Education, University of Melbourne, Melbourne, VIC, Australia; ^4^Basic College, Zhejiang Pharmaceutical Vocational University, Ningbo, China

**Keywords:** commognitive responsibility shift, collaborative problem solving (CPS), computer-supported, mathematics, one-to-one tutoring

## Abstract

The aim of this study is to use a commognitive responsibility framework to visualize responsibility shift in collaborative problem solving (CPS) during computer-supported one-to-one tutoring. Commognitive responsibility shift means that individuals’ cognitive responsibility shift can be reflected by the discourse in communication. For our sample, we chose a 15-year-old Chinese boy and his mathematics teacher with 6 years of teaching experience, both of whom have experienced computer-supported learning and teaching mathematics, respectively. We collected four tutoring videos (each 60–90 min; in total, more than 330 min) online, and a 45-min interview video from the teacher. We found that the third type of commognitive responsibility shift in both the teacher’s and student’s CPS behavior online is not only teacher–student comparison but also alternating-led, which includes teacher–student-led (TS) and student–teacher-led (ST).

## Introduction

With advanced technology tremendously transforming people’s way of life over decades, advanced information and communication technologies (ICT) have developed rapidly, leading to many new computer applications, such as e-mail, chat rooms, video conferencing, simulations, and discussion forums (Janssen et al., 2007). The relationship between technical development and education is a reciprocal one, where education always stands in relation to those skills, competencies, and techniques that are anticipated as necessary in a technological future (Rahm, 2021). Computer-supported online one-to-one tutoring is an extended form of online instruction based on the communication and cognition interaction between the teacher and the student (Humphry and Hampden-Thompson, 2019). It is increasingly popular due to its individualization and effectiveness (Weston and Bain, 2010), especially with the shift in the way of teaching from a traditional classroom setting to various online teaching platforms against the backdrop of COVID-19 pandemic in many countries.

To teach online, teachers require knowledge of online pedagogy. The principles of an online pedagogy include: (a) students should be responsible for their learning as they search for materials and build their knowledge and (b) students should collaborate and interact while working on projects (Pelz, 2010). Learning is still an individual process, but is influenced by group and inter-personal interactions (Kaye, 1992). Collaborative problem solving (CPS) in online environments can afford high levels of interactions among learners which can serve to develop critical thinking skills (Manathunga and Hernández-Leo, 2015). To our knowledge, there are fewer researches on the situation of CPS based on teachers and students in mathematics education. Our previous study based on Sfard’s (2008) commognitive framework, which suggests that individual cognition can be reflected by the discourse in communication, has found that the commognition of the teacher and the student in a series of one-to-one online tutoring courses can develop as the sequence of three types of processes, but not focusing on the CPS episodes.

In this study, we focus on the visualization of CPS periods in one-to-one computer-supported online tutoring to investigate commognitive responsibility shift episodes and their characteristics in CPS.

## Research Review

Our research is based on Sfard’s (2008) commognitive theory; therefore, in this section, we review her commognitive theory, while referring to the commognitive frontier literature, to provide a diverse perspective for this research. Then we review some interrelated researches about CPS because we explore the commognitive processes and responsibility shift in CPS.

### Commognitive Theory

Sfard’s theory of communicative cognition (Sfard, 2008) emphasizes the unity of thought process and communication process, which means that communication and thinking are two aspects of an activity called communicative cognitive activity (Supardi, 2021), while serving as the beginning of a complex process of constructing discourses of objective thinking, focusing on both social and individual aspects of thinking and learning. Communicative Cognitive Theory questions Descartes’ dual ontological epistemology of body and schizophrenia (Sfard, 2020). It is proposed that discourse in communication is the mirror of thinking (Lu et al., 2019), and discourse becomes the main object of communication cognitive theory (Martín-Molina et al., 2020). Considering discourse analysis as a fundamental research method and discourse learning and discourse development as conceptual tools for analyzing teaching activities (Bebell and O’Dwyer, 2010), the development of mathematical discourse can be conceived at the object level or the meta-level (Sfard, 2020; Shinno and Fujita, 2021), which is a breakthrough and innovation in research methods in the field of social culture. At the same time, the theory distinguishes two kinds of learning: (a) object-level learning and (b) learning at the meta level (Cooper and Lavie, 2021). Adopting a cooperative problem-solving evaluation framework and based on the concept of Vygotsky’s ZPD (zone of proximal development), the international ACT21S evaluation project proposed an innovative social and cognitive observation framework for teachers to use in the classroom (Woods et al., 2015). According to the cognition efficiency of students’ cooperative problem-solving process, it can be divided into six stages: (a) exploration, (b) systematic trial and error correction, (c) information collection, strategic planning and execution, (d) effective work, (e) accurate strategic application, and (f) problem-solving.

Based on Bloom’s cognitive classification theory and commognition theory, our previous study developed a commognitive classification framework in which we found that mathematics commognition processes can be divided into six levels: (a) macroscopic pre-level, (b) knowledge and skill target level, (c) knowledge level, (d) skill level, (e) research, and (f) evaluation level. After the visualization of the cognitive communication, we divided teacher–student commognition in the online teacher–student one-to-one tutoring episode into three situations: (a) teacher-led commognition, (b) comparison of commognitive processes, and (c) student-led commognition. Student-led commognition is likely to be more conducive to knowledge construction and learning.

### Collaborative Problem Solving

According to Assessment and Teaching of the 21st Century Skills (ATC21S), the construct of CPS was described as a combination of critical thinking, problem solving, decision making, and collaboration. It was argued that these skills could be conflated into a single complex set of skills under the title CPS (Care et al., 2016).

Student characteristics such as interpersonal skills (IPS), attitudes, emotions (Järvenoja and Järvelä, 2009), personality factors (e.g., “Big Five” factors, namely, openness, conscientiousness, extraversion, agreeableness, and neuroticism) and motivation (Gomez et al., 2010) can all affect individual and CPS success (Morgeson et al., 2005; O’Neill et al., 2012). IPS and the attitudinal, behavioral, and cognitive components are also considered critical components of performing effectively in collaborative situations. IPS has been described as a form of social perception and social cognition involving processes such as attention and decoding in interpersonal situations. IPS can be likened to a form of social intelligence, involving knowledge of social customs, expectations, and problem solving (McDonald and Klein, 2003). Further, it rests on an “ability to understand” behavior, cognition, and attitudes of individuals (including oneself) and to translate understanding into appropriate behaviors in social situations (Marlowe, 1986).

## Research Design

In this section, we introduce our research design. This section articulates our study question, study environment, and the coding principle of CPS in the online one-to-one tutoring.

### Study Questions

The research mainly focuses on the visual presentation of the commognitive processes and their characteristics in CPS of the one-to-one online tutoring process between one teacher and one student. We focus on the following questions:

1. In an online one-to-one mentoring process, how can we code CPS based on the dialog?
2. What different commognitive responsibility shift types of the CPS can be visualized between teacher and student in an online one-to-one tutoring episode?
3. What features and categories of commognition in different commognitive responsibility shift types of CPS can be visualized between teacher and student in an online one-to-one tutoring episode?

### Lesson Samples

Taking into consideration that a moderate level of cognitive conflicts may be more representative, we chose a Year-10 senior high student from Fujian Province in China and a Chinese teacher with 6 years experience of teaching high school mathematics as our two participants. The student is judged by the teacher to be of medium ability with regard to the class group and participates actively in communication with the teacher.

The four lesson samples were recorded in July 2018. The lessons involve the introduction and application of set theory, which is from the first chapter of Compulsory 1 of senior high school mathematics published by People’s Education Press of China (PEP) and training several key ideas studied by students at this level, like the discussion on classification and combination of number and shape. In detail, the first session focuses on the meaning and representation of sets, and the course duration is 62 min. The second session is the main explanation of the basic relationship between sets and related applications lasting 98 min long. The basic operation of sets is divided in two parts: Sessions 3 and 4. Session 3 introduces the first part of sets operation with a duration of 83 min and Session 4 the second that of 96 min.

According to our previous studies, the characteristics of commognition process and commognitive responsibility of each session are listed as follows: (a) The first session is a typical sample lesson of teacher-led commognition process, where the teacher mainly held commognitive responsibility. (b) The second session is the comparison of commognitive processes between student and teacher with an alternating shift in the commognitive responsibility between the teacher and the student, and the student sees a quick increase in commognitive responsibility. (c) In the third session, the student leads the commognitive process. In Session 4, the student tends to dominate the process of commognition, which is a more typical process of online one-to-one teaching between a teacher and student. In both sessions, share sub-processes where student-led sub-processes dominated.

The examples in Tables 1, 2 list the discourses between the teacher and the student in online one-to-one tutoring, which are extracted from four-lesson samples as typical cases of different types of CPS. It is noteworthy that in Chinese mathematics curriculum, solving a problem may require multiple strategies and involve various mathematical perspectives. Therefore, in the four-teaching sessions described here, the language of sets is applied to other topics, such as coordinate systems, points of intersection and so on, as shown in the examples listed in Tables 1, 2.

**TABLE 1:** Coding principle of CPS of computer-supported one-to-one tutoring.

Stages	Categories	Example
Beginning	Showing the problem	*Teacher*: What are the elements of a set of natural numbers less than 4?
	Asking the problem	*Student*: What is empty set? Should the empty set be written?
Conclusion	Solving the problem	*Student*: The answer is *B*. *Teacher*: That’s it?
	Summarizing the problem	*Teacher*: You should pay all your attention to *Z* (Integer notation), and ignore the symbols in the middle, so you can’t forget to look at the symbols in the middle when you’re solving a problem.
	Shelving the problem (ST-N)	*Student*: I am still confused about the problem in the last class and haven’t got your explanation yet. *Teacher*: I forgot to prepare. Let me write it down, and we’ll talk about it in the following class.

**TABLE 2:** The commognitive responsibility shift types of CPS.

Types	Description	Example	
Teacher-led (T)	The collaborative problem solving is led by the teacher.	*Teacher*: Find the set of points in the second quadrant of a plane rectangular coordinate system.	
		*Student*: Well…	
		*Teacher*: If the answer doesn’t come up in two seconds, you should draft immediately.	
		*Student*: A plane rectangular coordinate system is…	
		*Teacher:* Make a draft and write as much information as possible on the draft paper.	
		(The student began to make a draft, and successfully got the answer with the help of the teacher.)	
Student- led (S)	The CPS is led by the student.	*Student:* Can an empty set be a subset, but not an element?	
		*Teacher*: An empty set can be a subset of any set.	
		*Student:* But it cannot be an element, right?	
		*Teacher*: It can also be an element, like set *C* = (Gonzalez-Gomez et al., 2020),	
		in this way an empty set can be an element as well.	
		*Student:* Only when it is written can it be a subset?	
		*Teacher:* Yes, an empty set is a subset only when it is written in a set.	
Alternating-led (A)	The CPS is led by both the teacher and the student.	Teacher–student–led (TS)	*Student:* I’ve got the answer. It is $\frac{7}{5}$*. *Teacher: $\frac{7}{5}$* alone? Nothing else? *Student:* What else? *Teacher:* Please note that the question asks the intersection consisting of a set. Think twice: What is the intersection? *Student*: Oh, I got it, the value of *y* is also required; *y* equals $\frac{2}{5}$, so the answer is $\frac{7}{5}$,$\frac{2}{5}$. *Teacher*: That’s all? *Student*: We should also add parentheses. Then it’s ($\frac{7}{5}$,$\frac{2}{5}$). *Teacher*: You are right.
		Student–teacher-led (ST)	*Teacher*: What are the elements of a set of natural numbers less than 4? *Student*: 0, 1, 2, 3. *Teacher*: Right, the set *A* = (Son and Lee, 2020) includes 4 elements, and the set *B* = (Jopling, 2012) also 4 elements, which shows the randomness of the set. *Student*: It also tells us about the reciprocity of sets. *Teacher*: Good job! It also reveals the reciprocity of sets.

*The fractions are the answer of the problem: “Find the set of intersection points of two first-order functions.”

### Online Environment

**FIGURE 1:**
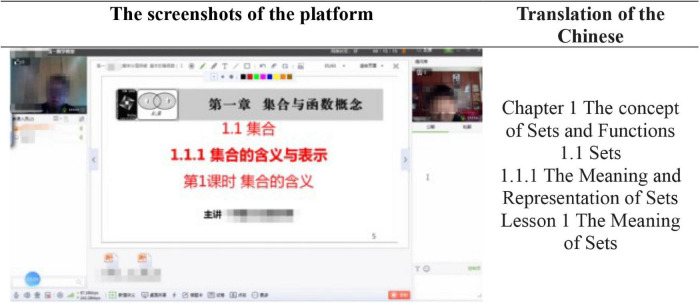
Screenshots of the platform and its translation in English.

The lesson samples are online tutoring videos recorded by computer-supported one-to-one tutoring platforms, which include online tutoring demonstration platform (Figure 1). As shown in Figure 1, the online tutoring demonstration platform consists of three major parts: (a) the window of the teacher on the left, (b) the window of the student on the right, and (c) the window of the courseware PPT, which also serves as a whiteboard in the middle. It enables the users to communicate either in dialog boxes through text or by the microphone.

### Coding Principle of Collaborative Problem Solving and Its Commognitive Responsibility Shift Types

**FIGURE 2:**
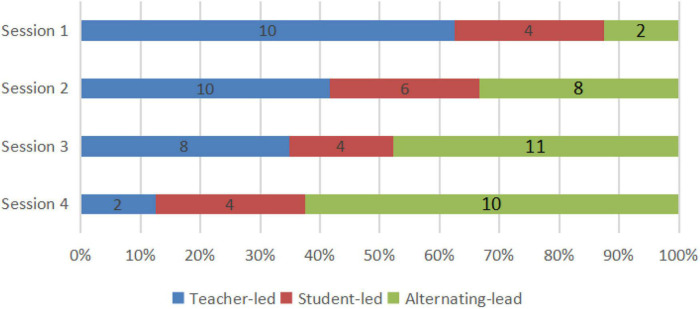
The sum of time periods for discussions of problem solving.

**FIGURE 3:**
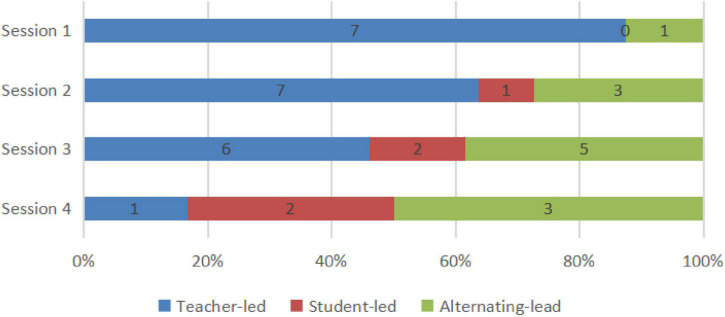
The sum of time periods for different kinds of CPS.

**FIGURE 4:**
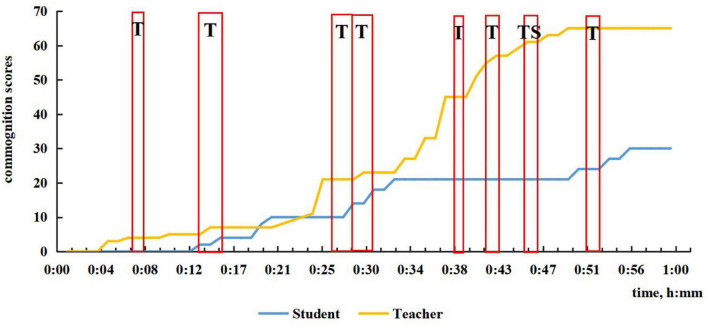
The time period and commognitive responsibility shift of CPS in Session 1.

**FIGURE 5:**
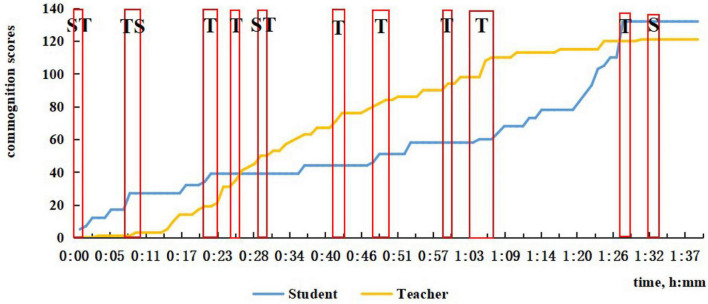
Time periods and commognitive responsibility shift of CPS in Session 2.

In our study, the coding principle of CPS is twofold. The first aspect is about the stages including beginning, and the conclusion of CPS (Table 1). The second aspect is about different categories of every stage of CPS (Table 2). In Table 1, the beginning stage is mainly led by the teacher to show the problem, and the student can also lead CPS by raising questions. The conclusion mainly includes three distinct ways: (a) the summary of the problem, (b) the solution of the problem, and (c) shelving the problem. By shelving the problem, we mean the problem was postponed, and the teacher and the student reached an agreement to discuss it at a later stage. If we define the first message that the student or the teacher exchanges as the initial state, and the subsequent one as meaningfully interrelated to it, then we can mark the period of CPS with the help of sequential analysis (denoted by red boxes in Figures 2–5). The examples listed in Tables 1, 2 are distracted from four-lesson samples as typical cases of different types of CPS.

Built upon the foundation of our previous studies, Table 2 is a more detailed and specific research on the discourses between the teacher and the student leading to CPS. Through the visualization of four-sample videos, we classified the CPS episodes between the teacher and the student into three types: (a) teacher-led, (b) student-led, and (c) alternating-led. The examples belonging to each type were distracted from four-sample tutoring sessions. In the teacher-led CPS, the teacher asked the student problems in PPT or designed one. In face of the student’s hesitation and perplexity, the teacher instructed the student either with problem-solving strategies or understandable questions. By following instructions, the student solved the problem in collaborative way. In the student-led CPS, the student took charge of the CPS episodes, putting forward a major question with sub-questions to resolve confusion. In such case, the teacher contributed to problem solving, instead of leading. Alternating-led CPS is the combination of the characteristics of teacher-led and student-led CPS by both the student and the teacher, in which they shared the leadership of CPS. We divided this type into two categories: (a) Teacher–student-led (TS) and (b) student–teacher-led (ST), to identify which side initiates the CPS.

Three researchers participated in the CPS coding process, and the coding Kappa coefficient of the results was 0.89, which was appropriate. As to inconsistent coding results, we reached a consensus after discussion and made necessary revisions.

## Results

In our previous research, we coded the discourses between the teacher and student after visualization from 0 to 5 and added up the commognition scores and plotted the sum sequentially according to the time sequence. The curve of the teacher and the student, respectively, reflects their commognitive level.

### Overview of Collaborative Problem Solving Episodes

In Figure 2, we located the time periods for discussions of problem solving in the four-lesson samples, counted the number, and calculated the percentage of different types of problem solving. Among these problem-solving discussions, CPS episodes were picked out after visualization using our coding principle. With the reference to the first question in our study’s purpose, we, respectively, presented number and percentage of different kinds of CPS episodes (Figure 3).

From the two figures, it can be seen that not all problem-solving episodes between the teacher and the student directly lead to CPS, which is evident from the number of problem solving and CPS. Communication which we have not classified as CPS is evident in three types of discussions and commognition between the teacher and the student, especially in student-led discussion and comparison discussion. As shown in Figure 3, in each session the number of each type of CPS was less than that of discussions of problem-solving. Discussions led by the student or by comparison would easily see a transfer to teacher-led CPS or fail to satisfy the coding principle to be CPS, if the teacher did not consciously step up guidance and use more incentives. Also, it is noticeable that the percentage of teacher-led CPS decreases in turn from Session 1 to Session 4, which echoes the finding of our previous study that the commognitive responsibility of the teacher was gradually released in the series of computer-supported one-to-one tutoring sessions. What is more, even though many student-led as well as comparison discussions were eliminated, we can witness a mild increase in student-led and alternating-led CPS from Session 1 to Session 4, as student-led commognition level gradually increases.

### Teacher-Led Collaborative Problem Solving Episodes

In the first session, since it was the student’s first math class in high school, the teacher sensed the student’s unfamiliarity and non-proficiency in solving high school math problems, so the teacher tended to take the commognitive responsibility and lead the commognitive process during the whole class. The teacher started the episodes of CPS when finding the student was unable to deal with the problem, or he pointed out the student’s errors. To achieve the learning goals, the teacher helped the student get the right answer by employing problem decomposition by asking questions that were more readily understandable. As shown in Figure 4, there is little CPS in the first half of Session 1, and minimal participation by the student in the latter part of the lesson.

### Alternating-Led Collaborative Problem Solving Episodes

#### Student–Teacher-Led Collaborative Problem Solving Episodes

**FIGURE 6:**
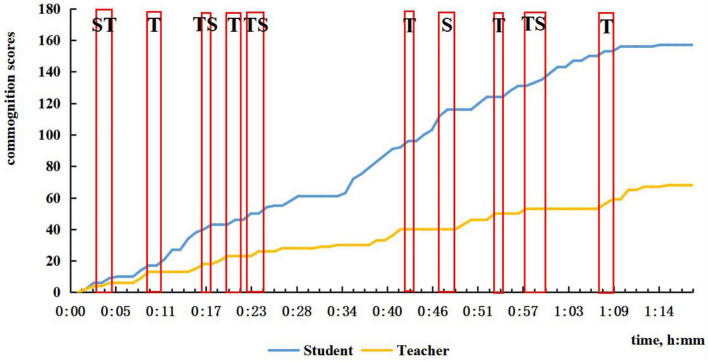
Time periods and commognitive responsibility shift of CPS in Session 3.

The characteristic of ST CPS episodes is that the sum of ST is greater than TS, and teachers typically need to facilitate, as shown in Figure 5 for Session 2. As shown in Figure 5, there are two ST CPS. This situation will also be a challenge for teachers. At the start of Session 2, the student said that he was confused about a problem that was left over, and the teacher could not solve it immediately, so it was agreed to discuss it next time. We also found that when it comes to discussion about homework, ST was more likely to occur because when the student did not know why his answer was wrong. He would then cast doubt in the light of his understanding at the beginning of Session 2 and Session 3 (Figure 6). Also, from 29 to 30 min in Session 2, the solution to the problem contained the answer bur was incomplete and not accepted by the teacher. The student followed the teacher’s tips and simple questions like “What’s next?,” “That’s all?,” “Think twice,” etc., and corrected previous answer. With simple feedback, the student was motivated to cope with the problem himself.

#### Teacher-Student Led Collaborative Problem Solving Episodes

The characteristic of TS CPS episodes is that the sum of TS is greater than ST, as shown in Figure 6 for Session 3. A typical teacher-led CPS occurred in Session 1 from 45 to 46 min, when the student supplemented the teacher’s summary to the teacher’s delight and surprise (see the example in Table 1). Another typical case is when the student was perplexed by the teacher’s explanation, he raised rhetorical questions requiring the teacher to interpret in a more detailed, specific way by presenting examples, drawing geometries, etc. As shown in Figures 4, 6, there is one teacher–student-led (TS) CPS, in Session 1 and three such episodes in Session 3.

### Student-Led Collaborative Problem Solving Episodes

**FIGURE 7:**
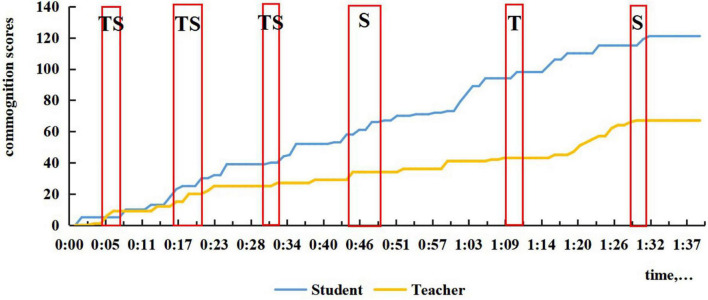
The time period and commognitive responsibility shift of CPS in Session 4.

From Session 2 to Session 4, the frequency and duration of student-led CPS increased. In the second session, when the teacher mentioned the concept of empty set, the student asked its meaning and entailed the teacher’s explanation of whether it should be written. In Session 3 starting from 47 min, while the teacher was introducing empty set, the student linked the concept of empty set with the ideas of subset and element, asking how to distinguish between the three concepts. After the teacher explained that an empty set can be a subset of any set, the student proposed a further question “It cannot be an element?” showing a deeper comprehension of empty set. The first student-led CPS in Session 4 lasts for the longest time, from 45 to 48 min when new problems were presented, and the student noticed a fine detail in problem expression, asking why there was no bracket after the ∈in the relation *a*∈ *a ∩* (*C* ∪ *B*). The teacher knew the student mixed up the functions of belonging sign, intersection sign, and union sign, so the teacher drew a conclusion after a series of questions from the student. The second student-led CPS occurred toward the end starting from 92 min in doing reviewing exercises, and the student was encouraged to solve a difficult problem. While calculating, the student outlined every step to ensure the correctness with simple yes or no feedback from the teacher. During this episode, the students led the CPS and resolved the problem. As Figure 7 shows, in the later period of the class when new problems were presented, the student-led CPS lasted longer than the teacher-led CPS.

## Discussion

The coding principles adopted in our research into the visualization of CPS episodes in computer-supported one-to-one tutoring have built upon our previous research and confirm its validity and practicability in describing the three types of CPS episodes following the commognitive level of the teacher and the student. Since the discourses leading to CPS between the teacher and the student in online tutoring are visualized, the coding framework could be further extended to computer-supported one-to-many tutoring, in which the students can be labeled as S1, S2, etc.

Across the four sessions, there is a significant variation in the frequency and duration of different CPS types. Teacher-led CPS episodes primarily took place when examples of problems were presented after the introduction of knowledge to assist the student’s consolidation. The frequency of teacher-led episodes in every session suggests that the work of the tutor is among the most important aspects that are proposed as determining factors in the success of the training in this type of training (Jiménez et al., 2017).

However, from the first session to the third session, the incidence of teacher-led CPS gradually decreased, and teacher–student-led and student-led CPS were seen to be increasing as the student’s commognitive level appears to rise. The decrease in teacher-led CPS episodes suggests that the teacher relinquished the dominance of CPS during the class to the student. The demonstration of a problem was no longer completed by the teacher alone but required the student’s more active participation in problem solving and seemed to rely on the student taking more initiative. There were more opportunities for the student to put forward his views toward the problem, explaining the increase in teacher-student-led CPS. During these episodes, the student and the teacher shared and shifted the leadership of CPS, with both of their discourses being of equal importance in CPS. In the student-led CPS, the student took charge of a CPS episode as he came up with self-initiating questions through independent thinking and became more active in class participation. In this stage, the teacher undertook a greater listening and less leading role.

The visualization of CPS episodes in online one-to-one tutoring and their features and categories of the commognition in different types of CPS can serve as guidance for both teachers and students. Teachers can design and modify the tutoring strategies in online one-to-one tutoring, concretely perceive teacher-led CPS episodes. For example, when teachers feel that most of the CPS episodes in the class is being led by themselves, they need to shift direction allowing students to contribute more to problem-solving and lead more CPS episodes. By using our study for training, it is possible for teachers to adjust tutoring strategies at a certain point in time to give students greater initiative to solve certain problems and guide them to make more concerted endeavors in problem solving, upgrade teachers’ beliefs about or conceptions of mathematical problem solving (Son and Lee, 2020). For students, the visualization of CPS can influence their participation. When participation of group members can be visualized, this helps to identify the contribution of each group member; establishing a link between a group member and his or her contribution to the collaboration (Jermann and Dillenbourg, 2008). Likewise, in online tutoring, this evaluation provides students with motivational incentives to invest effort into collaboration.

## Conclusion

The analysis of the processes and elements of computer-supported collaborative learning (CSCL) constitutes a key research direction in education (Munoz-Carril et al., 2021); however, most studies focus on collaborative learning outcomes rather than collaborative learning processes (Zheng et al., 2021). In this study, we built a framework of the coding principles serving to visualize CPS between the teacher and the student in a computer-supported one-to-one tutoring. We hope that in the future when voice recognition and artificial intelligence (AI) technology have reached a certain level, our coding principle can serve as a reference for AI to visualize the episodes of CPS in online circumstances. The AI precision education model can promote students’ learning experience and improve their academic performance (Lin and Lai, 2021). This coincides with the concept of AI-assisted learning. AI can realize real-time identification, capture, and realization of CPS of teachers and students in a visual network environment, providing technology for teachers to further optimize teaching strategies and pay close attention to students’ cognitive changes and fluctuations. In addition, Educational Data Mining (EDM) can be considered an innovative tool or technique to provide new insights into teaching and learning (Shin and Shim, 2020).

With the help of the coding framework, we found that the commognitive processes of CPS in online one-to-one tutoring can be divided into three types: Teacher-led, student-led, and alternating-led. To figure out who triggered the CPS from the start, we classified alternating-led into two categories: TS and ST depending on who assumed a greater proportion of the lead. Some important features of the commognition in different types of CPS are as follows:

(1) The teacher-led CPS decreases in number and duration as the teacher’s commognition level reduces and gradually giving away commognitive responsibility.
(2) Where commognitive responsibility was gradually released from the teacher to the student and a more evenly shared alternating condition emerged where the teacher and student lead the CPS episodes together.
(3) Student-led CPS sees a pronounced increase in the student’s opportunities to lead and to contribute directly to the commognition.

By and large, the features of the commognition in CPS episodes coincide well with the features of commognition episodes and commognitive responsibility shift between the teacher and the student. Nevertheless, there is a delineated boundary with two major differences: (1) Student-led CPS is not necessarily the consequence of higher commognition level of the student. Regardless of the student’s escalating commognition level, the student-led CPS is witnessed a slow growth; (2) The teacher sticks to the role of the “critical variables” (Cabero, 2006) and is mainly responsible for series of session, even when his/her commognition level has been far exceeded. From Session 1 to Session 4, a shift in the role of the teacher from a director to a participant is shown in Figure 3, but the teacher still retains the commognitive responsibility and leads the CPS episodes. As a result, the number of ST and Student-led CPS is relatively small. This may be a universal phenomenon in Chinese teachers’ tutoring both online and in classroom, especially mathematics teachers’ tutoring. However, the occurrence of ST and student-led CPS in online one-to-one sessions indicates important characteristics of student-led CPS.

In the future, the online tutors ought to manage more and longer ST and student-led CPS in online one-to-one tutoring to create online lessons that are more efficient and student-centered. Accordingly, we propose the following suggestions for online tutors: (1) decentralizing responsibility in online sessions to grant more participation of students in problem-solving; (2) utilizing the process of commognition to form effective CPS with incentives and stimuli, especially, when the CPS episodes is led by students.

Our study has some limitations. Though we have divided the episodes of CPS in computer-supported one-to-one tutoring into three types based on the coding principle, and this study does not explore further into the specific collaborative activities in each type. Additionally, since this study mainly focuses on verbal discourses between the teacher and one student, further studies on the multimodal learning including gesture sensing, infrared imaging, and eye-tracking in computer-supported one-to-one tutoring are needed to provide a deeper insight into the minute-by-minute development of several activities, especially when they involve multiple dimensions of interaction and social interaction (Blikstein, 2013). On the foundation of current researches, we will carry out further study on this topic in the following research work. In more realistic classroom settings involving CPS with groups of students, it is feasible and desirable to expand the methodology used in this study to include not only commognitive shifts between teacher and students but also among students themselves.

## Data Availability Statement

The raw data supporting the conclusions of this article will be made available by the authors, without undue reservation.

## Ethics Statement

The studies involving human participants were reviewed and approved by the Hangzhou Normal University Ethics Committee. The patients/participants provided their written informed consent to participate in this study. Written informed consent was obtained from the individual(s) for the publication of any potentially identifiable images or data included in this article.

## Author Contributions

JL and PT designed this study and drafted the original manuscript. RF and ZS collected the data. JL, PT, RF, MS, MZ, and ZS had full access to the data and analysis. ZS finalized the data analysis results. All authors reviewed critically, provided feedback on the initial version of the manuscript, and approved the final version of the manuscript.

## Conflict of Interest

The authors declare that the research was conducted in the absence of any commercial or financial relationships that could be construed as a potential conflict of interest.

## Publisher’s Note

All claims expressed in this article are solely those of the authors and do not necessarily represent those of their affiliated organizations, or those of the publisher, the editors and the reviewers. Any product that may be evaluated in this article, or claim that may be made by its manufacturer, is not guaranteed or endorsed by the publisher.
